# Case Report: A Case Study Documenting the Activity of Atezolizumab in a PD-L1-Negative Triple-Negative Breast Cancer

**DOI:** 10.3389/fonc.2021.710596

**Published:** 2021-09-20

**Authors:** Fara Brasó-Maristany, Miriam Sansó, Nuria Chic, Débora Martínez, Blanca González-Farré, Esther Sanfeliu, Lucio Ghiglione, Esther Carcelero, Javier Garcia-Corbacho, Marcelo Sánchez, Dolors Soy, Pedro Jares, Vicente Peg, Cristina Saura, Montserrat Muñoz, Aleix Prat, Ana Vivancos

**Affiliations:** ^1^Translational Genomics and Targeted Therapies in Solid Tumors, August Pi i Sunyer Biomedical Research Institute (IDIBAPS), Barcelona, Spain; ^2^Department of Medical Oncology, Hospital Clínic of Barcelona, Barcelona, Spain; ^3^Cancer Genomics Group, Vall d’Hebron Institute of Oncology, Barcelona, Spain; ^4^Department of Oncology and Hematology, Health Research Institute of the Balearic Islands (IdISBa), Palma de Mallorca, Spain; ^5^Department of Pathology, Hospital Clínic de Barcelona, Barcelona, Spain; ^6^Department of Pharmacy, Hospital Clínic of Barcelona, Barcelona, Spain; ^7^Department of Radiology, Hospital Clínic of Barcelona, Barcelona, Spain; ^8^Molecular Biology Core, Hospital Clinic of Barcelona, Barcelona, Spain; ^9^Vall d’Hebron University Hospital and Vall d’Hebron Institute of Oncology (VHIO), Medical Oncology Service, Barcelona, Spain; ^10^SOLTI Cooperative Group, Barcelona, Spain; ^11^Department of Oncology, Institut Oncològic Baselga (IOB) Institute of Oncology, Quironsalud Group, Barcelona, Spain; ^12^Department of Medicine, University of Barcelona, Barcelona, Spain

**Keywords:** immunotherapy, breast cancer, biomarkers, ctDNA, case report

## Abstract

The immune checkpoint inhibitor atezolizumab is approved for PD-L1-positive triple-negative breast cancer (TNBC). However, no activity of atezolizumab in PD-L1-negative TNBC has been reported to date. Here, we present the case study of a woman with TNBC with low tumor infiltrating lymphocytes and PD-L1-negative disease, which achieved a significant response to atezolizumab monotherapy and durable response after the combination of atezolizumab and nab-paclitaxel. The comprehensive genomic analysis that we performed in her tumor and plasma samples revealed high tumor mutational burden (TMB), presence of the APOBEC genetic signatures, high expression of the tumor inflammation signature, and a HER2-enriched subtype by the PAM50 assay. Some of these biomarkers have been shown to independently predict response to immunotherapy in other tumors and may explain the durable response in our patient. Our work warrants further translational studies to identify biomarkers of response to immune checkpoint inhibitors in TNBC beyond PD-L1 expression and to better select patients that will benefit from immunotherapy.

## Introduction

Triple-negative breast cancer (TNBC) lacks expression of estrogen receptor (ER), progesterone receptor (PR), and the human epidermal growth factor receptor 2 (HER2); accounts for 15%–20% of all breast cancers; affects young women; and is highly aggressive. While targeted therapies are available for ER-positive (ER+) and HER2-positive (HER2+) breast cancer, chemotherapy remains the standard of care for TNBC. Among the different subtypes, TNBC is the most immunogenic and has the highest median number of tumor-infiltrating lymphocytes (TILs), PD-L1 expression, and tumor mutational burden (TMB), all of which are associated with immune activity (1). In this context, immunotherapy with atezolizumab, an anti-PD-L1 drug antibody, has been approved for PD-L1-positive (PD-L1+) (i.e., ≥1% PD-L1+ tumor-infiltrating immune cells) advanced TNBC in combination with nab-paclitaxel (2). On the other side, activity of pembrolizumab monotherapy in patients with pre-treated metastatic breast cancer with high TMB has recently been reported (3). However, no activity of immune checkpoint inhibitors in PD-L1-negative TNBC has been observed to date, and the predictive value of TMB beyond PD-L1 expression is still unknown.

Here, we describe a case of a woman with an initial diagnosis of HER2+ localized tumor treated with curative therapy that relapsed 9 years later being an ER+/HER2-negative metastatic breast cancer. She progressed to first-line endocrine therapy and palbociclib, a CDK4/6 inhibitor, and whose tumor became then triple-negative. Molecular characterization of her metastatic TNBC observed absence of PD-L1 expression, but high TMB, presence of the Apolipoprotein B mRNA Editing Catalytic Polypeptide-like (APOBEC) genetic signatures, high expression of the tumor inflammation signature (TIS), and a HER2-enriched subtype by the PAM50 assay. Based on this tumor profile, Hospital Clinic Molecular Tumor Board indicated one cycle of atezolizumab followed by atezolizumab in combination with nab-paclitaxel. Plasma circulating tumor DNA (ctDNA) and radiological imaging were used to assess treatment efficacy. The patient presented in this report has given her consent for publication.

## Case Presentation

A 44-year-old white Spanish woman with no significant familiar or medical history was initially diagnosed with a left breast cancer in 2008 (pT2N3M0). The pathology report revealed an ER+, PR-positive, and HER2+ invasive carcinoma of the breast. She underwent surgery in October 2008 and received adjuvant anti-HER2-based chemotherapy, followed by locoregional radiotherapy and endocrine therapy.

In April 2018, the patient was diagnosed with right supraclavicular and axillary positive lymph nodes (17 mm and 3 mm) by ultrasound. Bone metastasis was detected by PET/CT scan. A core biopsy of the right supraclavicular lymph nodes was performed and revealed an ER+ and HER2-negative invasive lobular carcinoma. In this tumor biopsy, an amplicon-based DNA sequencing panel of pan-cancer genes showed the presence of a *PIK3CA* E545K (18% mutant allelic frequency [MAF]) and 726F (16% MAF) somatic mutations. As a first-line treatment, she received fulvestrant and palbociclib (125 mg daily, 3 weeks on, 1 week off) until May 2019 (13 months of treatment), when bone and lymph node progressions were observed.

Two new biopsies of the right breast and axillary node were performed and revealed a TNBC lobular carcinoma. In the breast lesion, the tumor had a Ki67 of 18% and less than 1% TILs and was PD-L1-negative by immunohistochemistry (Ventana PD-L1 antibody clone SP142). Intrinsic subtype by PAM50/Prosigna^®^ revealed a HER2-enriched subtype with low levels of *ERBB2* mRNA. A DNA sequencing panel of 431 genes showed *PIK3CA* E545K and *TP53* Q331* mutations, a high TMB of 38.5 mutations per megabase (mut/Mb) and an APOBEC-mutational profile, including signatures S2 and S13. Guardant360 74-gene panel confirmed the presence of multiple somatic mutations, including *PIK3CA* E545K mutation with a variant allele fraction of 12.2%.

**Figure 1:**
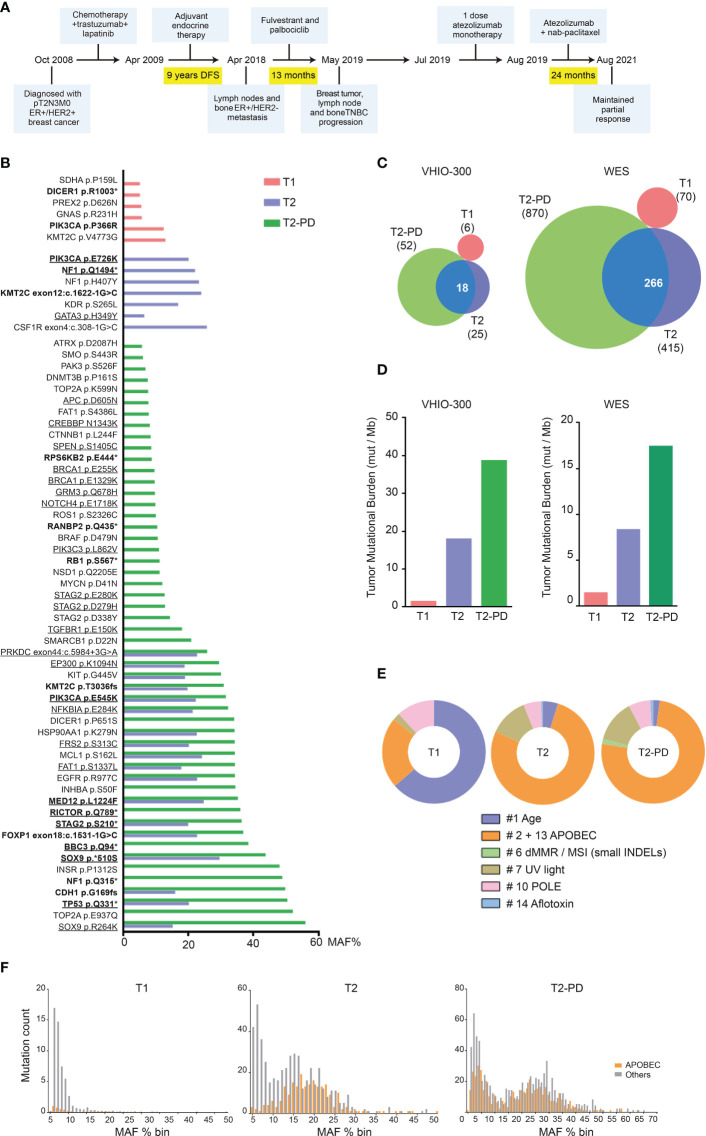
Patient treatment timeline and DNA alterations. **(A)** Patient treatment timeline. **(B)** Mutant Allelic Frequency (MAF; %) in T1 (sample of 2008), T2 (sample of 2018), and T2-PD (sample of 2019) determined using the VHIO-300 panel. Variants classified as pathogenic or likely pathogenic are highlighted in bold; variants matching APOBEC DNA-sequence signature are underlined. **(C)** Overlap of somatic mutations between T1, T2, and T2-PD using the VHIO-300 panel and whole-exome sequencing (WES). **(D)** TMB expressed as mutations/megabase in T1, T2, and T2-PD using the VHIO-300 panel and WES. **(E)** COSMIC mutational signatures of age, APOBEC defect (APOBEC), defective mismatch repair/microsatellite instability (dMMR/MSI) reflected as small insertions and deletions (INDELs), ultraviolet light (UV), polymerase E defect (POLE), and aflotoxin effect in T1, T2, and T2-PD determined by WES. **(F)** MAF distribution for APOBEC-related and other mutations in T1, T2, and T2-PD determined by WES.

Based on these results, the clinical case was presented at our weekly multidisciplinary Tumor Board at Hospital Clinic of Barcelona. Since activity of immunotherapy in patients with breast cancer with high TMB has been reported (3), a regimen of single-agent immunotherapy combined with chemotherapy was planned. More specifically, in July 2019, the patient received one dose of 1200 mg atezolizumab monotherapy and after 3 weeks continued with 1200 mg atezolizumab (day 1) plus weekly 100 mg/m^2^ nab-paclitaxel. In August 2021 (24 months of treatment), the patient continues on treatment presenting a maintained partial response and an excellent performance status. The treatment history is summarized in **Figure 1A**.

## Genomic Analyses

Analysis of the DNA from the three tumor specimens and a buffy coat blood sample by next-generation sequencing using the VHIO-300 capture-based panel of 431 pan-cancer related genes and whole-exome sequencing (WES) revealed an independent genetic origin of samples from 2008 and 2018, while the tumor from 2019 clearly was a progression (PD) of the 2018 lesion. Thus, samples were relabeled as T1 (breast sample, 2008), T2 (lymph node sample, 2018), and T2-PD (breast sample, 2019). Genomic analyses revealed a completely different mutational profile of T1 *versus* T2 and T2-PD; the vast majority of mutations in T2 were also present in T2-PD, while none of them were present in T1, and 34 somatic variants were found exclusively in T2-PD (e.g., *RB1* S567* and a nonsense *NF1* mutation in residue Q315*) (**Figures 1B, C**). In addition, TMB was low in T1 compared to T2 and T2-PD (**Figure 1D**). Analysis of COSMIC mutational signatures from the WES results showed a dominant pattern related to age in T1, while the high TMB in T2 and T2-PD was linked to a sequence context preference of cytosine mutations caused by APOBEC enzymes (4) (**Figure 1E**). Finally, a study of MAF distribution showed a clonal peak for APOBEC-related mutations in T2, with increased average MAF % in T2-PD, plus a second peak of subclonal mutations also linked to APOBEC defect in the T2-PD (**Figure 1F**).

**Figure 2:**
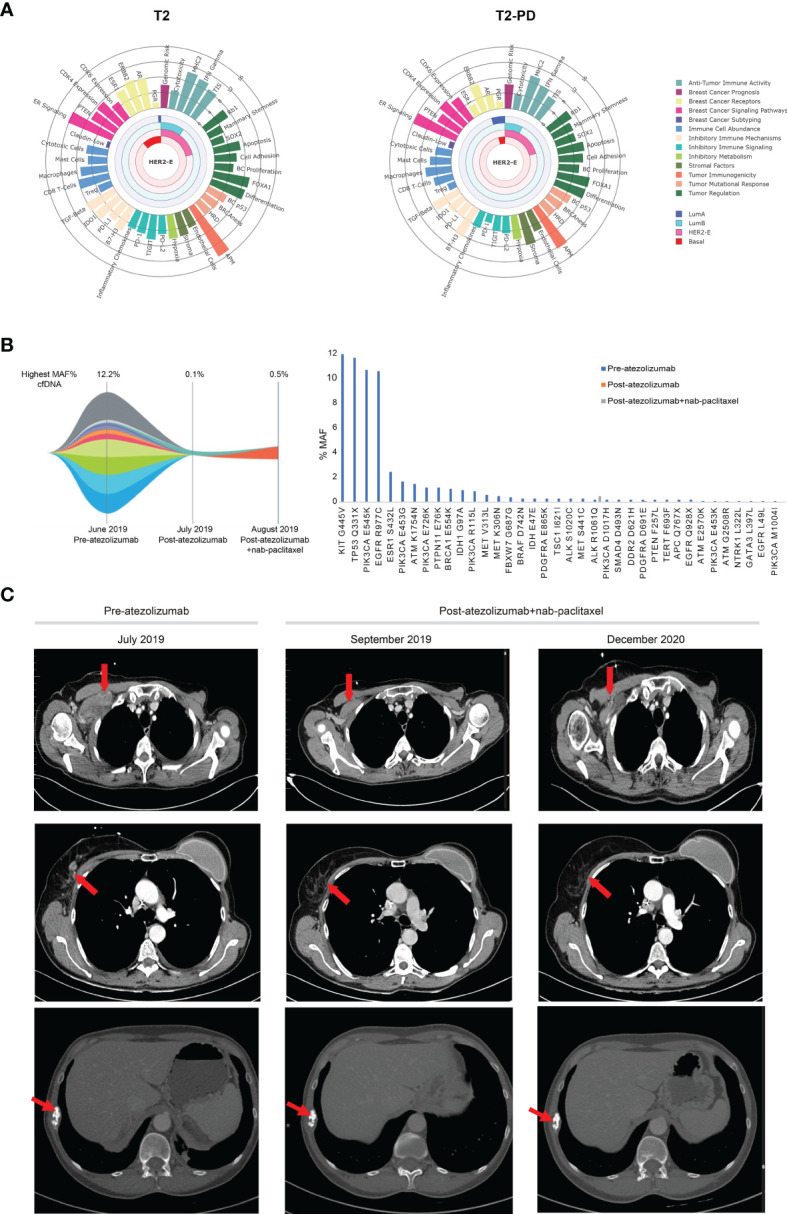
Gene expression and molecular and clinical response to atezolizumab and nab-paclitaxel. **(A)** The Wheel Plots depict the relative expression of each signature for T2 and T2-PD samples determined using the Breast Cancer 360 nCounter-based gene expression panel. Signatures are grouped based on the biological process in which they belong. The Luminal A, Luminal B, HER2-enriched, and Basal-like subtype correlation scores are shown as a radial arc. Signature scores (0–16, low to high) are represented as radial projections. **(B)** Guardant360 Tumor Response Map showing the highest variant allele fraction (%) and MAF (%) assessed in ctDNA using Guardant360 results before treatment, after 3 weeks of atezolizumab monotherapy, and after combining atezolizumab and nab-paclitaxel. **(C)** CT scan. Red arrows indicate lesions on the soft tissue of the chest wall (top) and a mammary node (middle) and bone (bottom) during treatment.

RNA from T2 and T2-PD were analyzed at the nCounter Breast Cancer 360 Panel. *ESR1* expression was decreased in T2-PD compared to T2, consistent with the immunohistochemistry results. Both T2 and T2-PD were classified as HER2-enriched and showed high expression of immune signatures (i.e., MHC-II, IFN-gamma, TIS, antigen presenting machinery) (**Figure 2A**). PD-L1 and PD1 mRNA expression was low.

Since ctDNA can be a surrogate of response to therapy and long-term outcome (5), liquid biopsies were collected before atezolizumab, after 3 weeks of atezolizumab monotherapy and after 3 weeks of atezolizumab plus nab-paclitaxel. Plasma samples were sequenced using the standardized Guardant360 assay. Mutations in 37 genes were identified in the plasma sample before immunotherapy and highest variant allele frequency (VAF) was 12.2%. After atezolizumab monotherapy, the only detectable mutation was *PDGFRA* D691E (VAF = 0.1%). After 1 month of atezolizumab plus nab-paclitaxel, the only mutation detected was *ALK* R1061Q (VAF = 0.5%) (**Figure 2B**), and after 2 months, a chest CT scan confirmed a partial response as observed on the soft tissue of the chest wall and a mammary node, and in December 2020, the patient continued in clinical and radiological response (**Figure 2C**). Bone metastasis was followed up by CT scan every 3 months, with stable disease as the best response.

## Discussion

Acquisition of genomic alterations and changes in gene expression profiles may lead to treatment failure and disease progression. Here, we report a patient diagnosed of ER+/HER2-negative metastatic breast cancer who progressed to first-line endocrine therapy in combination with CDK4/6 inhibition, and the progressive disease lost ER expression and became TNBC; it was PD-L1-negative and benefited from atezolizumab in combination with nab-paclitaxel. Although our study cannot identify the main cause of the patient’s response to atezolizumab alone and in combination with nab-paclitaxel, the extensive molecular characterization performed could provide clues about the features associated with immunotherapy benefit in PD-L1-negative TNBC.

The genomic analysis performed revealed that the pre-treatment ER+/HER2-negative tumors and the progression tumor samples after endocrine therapy and palbociclib had the following features: (1) high TMB, (2) presence of the APOBEC genetic signatures, (3) HER2-enriched, and (4) high expression of immune gene signatures such as TIS or interferon-gamma but not PD-L1 or PD1 mRNA. However, some molecular features were different between the two time points. For example, *NF1* and *RB1* mutations and lower expression of ER were identified in the progression sample, which also had a higher TMB score. Consistently, loss of ER and acquisition of *NF1* mutations have been associated with resistance to endocrine therapy (6), while acquisition of *RB1* mutations, APOBEC signatures, and the HER2-enriched subtype has been associated with resistance to palbociclib (7, 8). Moreover, the APOBEC genetic signatures have been previously associated with the HER2-enriched subtype and high TILs (9, 10). Indeed, APOBEC genetic signatures contribute to the acquisition of subclonal mutations, leading to genomic instability and potential neoantigens expression, which could induce immune response (11). The genetic origin of the APOBEC signatures in this case remains unknown. Among the possible explanations, we excluded germline loss of *APOBEC3B* (12). Unfortunately, we were not able to study the expression levels of *APOBEC3B* (13).

Currently, three biomarkers have been clinically validated as predictors of response to immune checkpoint blockade in TNBC [i.e., PD-L1 (2)] and across cancer types (i.e., TMB (3, 14) and mismatch repair deficiency (15). Indeed, the immunotherapy drug pembrolizumab for the treatment of patients with TMB-high tumors is approved by the Food and Drug Administration (FDA) (14). In TNBC, a modest benefit of immune checkpoint blockade has been observed in patients who are PD-L1-positive (2), which has led to the approval of the combination of atezolizumab plus nab-paclitaxel in PD-L1+ TNBC by the FDA. Other suggested predictive factors of response to immune checkpoint blockade include the TIS score (16), the APC signature (17), PD1 mRNA expression (18), and a T cell–inflamed gene expression profile (19). Some of these biomarkers have been shown to independently predict response to immunotherapy and may capture distinct features of neoantigenicity. Therefore, composite biomarkers may help better identify those patients that benefit to immune checkpoint inhibition and warrant further translational studies to improve patient selection.

To conclude, we present the case study of a TNBC with low TILs and PD-L1-negative disease, which achieved a significant response to atezolizumab monotherapy and durable response after the combination of atezolizumab and nab-paclitaxel, possibly explained by the APOBEC signatures, the high TMB, the high TIS score, and the HER2-enriched subtype, or the combination of several of these features. Moreover, the addition of chemotherapy could have helped turn the cold tumor microenvironment into hot, recruiting more T cells and improving response to atezolizumab (20). After 24 months, the patient continues in clinical and radiological response.

## Patient Perspective

After progression to first-line treatment (palbociclib + ET), the patient is really satisfied with a 24-month response to a well-tolerated scheme of atezolizumab plus weekly nab-paclitaxel with just alopecia G1 (she is using a cold cap to prevent it) and hepatotoxicity G2 that has been recovered just to delay treatment 1–2 weeks three times during these 2 years. She maintains an active life with ECOG 0.

## Data Availability Statement

The original contributions presented in the study are included in the article. Further inquiries can be directed to the corresponding authors.

## Ethics Statement

The studies involving human participants were reviewed and approved by Comité de Ética de la Investigación con medicamentos del Hospital Clínic de Barcelona. The patients/participants provided their written informed consent to participate in this study. Written informed consent was obtained from the individual(s) for the publication of any potentially identifiable images or data included in this article.

## Author Contributions

Experimental study design: FB-M, MiS, AP, and AV. Provision of study materials or patients: FB-M, MiS, NC, DM, BG-F, ES, LG, EC, JG-C, MaS, DS, VP, CS, MM, AP, and AV. Data analysis and interpretation: FB-M, MiS, NC, MM, AP, and AV. Writing of the manuscript FB-M, MiS, AP, and AV. Revision of the manuscript: FB-M, MiS, NC, DM, BG-F, ES, LG, EC, JG-C, MaS, DS, VP, P-J, CS, MM, AP, and AV. Supervision: AP and AV. All authors contributed to the article and approved the submitted version.

## Funding

This study has received funding from Instituto de Salud Carlos III—PI19/01846 (to AP), Breast Cancer Now—2018NOVPCC1294 (to AP), Breast Cancer Research Foundation-AACR Career Development Awards for Translational Breast Cancer Research 19-20-26-PRAT (to AP), Fundació La Marató TV3 201935-30 (to AP), the European Union’s Horizon 2020 research and innovation programme H2020-SC1-BHC-2018-2020 (to AP), Asociación de Cáncer de Mama Metastásico CMM_CHIARAG19_001 (to AP), Pas a Pas (to AP), Save the Mama (to AP), Fundación Científica Asociación Española Contra el Cáncer AECC_Postdoctoral17-1062 (to FB-M) and INVES19056SANS (to MiS), FERO-ghd 2020 breast cancer award (MS), and Generalitat de Catalunya Peris PhD4MD 2019 SLT008/18/00122 (to NC).

## Conflict of Interest

Potential conflicts of interest are the following: AP reports consulting fees from Nanostring Technologies, Roche, Pfizer, Novartis, AstraZeneca, Foundation Medicine, Guardant Health, and Daiichi Sankyo outside the submitted work. AV reports consulting fees from Sysmex, Novartis, Merck, Bristol Meyers Squibb, Guardant Health, and Incyte; research funding from Bristol Meyers Squibb; and royalties from Ferrer outside the submitted work. VP has received fees as consultant, participated in advisory boards, or received travel grants from Sysmex, Roche, MSD, AstraZeneca, Bayer, and Exact Sciences outside the submitted work. CS has declared personal fees as consultant and advisory board or travel grants of AstraZeneca, Daiichi Sankyo, Eisai, Exact Sciences, Exeter Pharma, F. Hoffmann–La Roche Ltd, MediTech, Merck Sharp & Dohme, Novartis, Pfizer, Philips, Piere Fabre, Puma, Roche Farma, Sanofi-Aventis, SeaGen, and Zymeworks and institutional financial interests from AstraZeneca, Daiichi Sankyo, Eli Lilly and Company, Genentech, Immunomedics, Macrogenics, Merck, Sharp and Dhome España S.A., Novartis, Pfizer, Piqur Therapeutics, Puma, Roche, Synthon, and Zenith Pharma. CS and AP are Board Members of SOLTI Cooperative Group and are employed by Institut Oncològic Baselga (IOB), Quironsalud Group.

The remaining authors declare that the research was conducted in the absence of any commercial or financial relationships that could be construed as a potential conflict of interest.

## Publisher’s Note

All claims expressed in this article are solely those of the authors and do not necessarily represent those of their affiliated organizations, or those of the publisher, the editors and the reviewers. Any product that may be evaluated in this article, or claim that may be made by its manufacturer, is not guaranteed or endorsed by the publisher.
